# *GenX 3*: the latest generation of an established tool

**DOI:** 10.1107/S1600576722006653

**Published:** 2022-07-30

**Authors:** Artur Glavic, Matts Björck

**Affiliations:** a Laboratory for Neutron and Muon Instrumentation, Paul Scherrer Institut, 5232 Villigen PSI, Switzerland; b Department of Physics, Uppsala Universitet, Box 530, SE-751 21, Uppsala, Sweden; Australian Centre for Neutron Scattering, ANSTO, Australia

**Keywords:** reflectometry, surface X-ray diffraction, neutron analysis, X-ray analysis

## Abstract

Since its publication more than 15 years ago the *GenX* software has been continuously developed and has established itself as a standard package for analyzing X-ray and neutron reflectometry data. The evolution of the software during the last two major revisions is reported here. This includes a simplified model builder for beginners, simple samples, additional sample models, statistical error analysis and the use of just-in-time compilation modules for the reflectometry kernel to achieve higher performance. In addition, the influence of experimental errors on the reflectivity curve is discussed, and new features are described that allow the user to include these in the error statistics to improve the fitting and uncertainty estimation.

## Abstract 2

Improvements to the *GenX* program are discussed, including performance, model building and error analysis.

## 1. Introduction

Neutron and X-ray reflectivity measurements often rely on modeling the sample structure to reproduce the experimental data. The process of building and subsequently refining that model and its parameters to give the best fit to the measurement is thus of great importance to the scientific community. In addition to countless small programs and scripts that circulate at universities and research institutes, there are several established and professionally maintained programs that focus on various aspects of science. One important application is the modeling of soft-matter structures using system-specific parameterization, for example in the programs *RefNX* (Nelson & Prescott, 2019 ▸) or *Refl1D* (Kienzle *et al.*, 2011 ▸). Other software focuses on simplified interactive online modeling (Maranville, 2017 ▸) or scripting (Koutsioubas, 2021 ▸), grazing-incidence scattering (Pospelov *et al.*, 2020 ▸), or resonant and magnetic X-ray modeling (Macke *et al.*, 2014 ▸; Vignaud & Gibaud, 2019 ▸). The original *GenX* (Björck & Andersson, 2007 ▸) software was a tool to perform that modeling and aid the process of finding the right model parameters by differential evolution (DE) refinement. To simplify its use and aid the user, a minimal graphical user interface (GUI) was included. While some recent studies require sophisticated and complex models combining X-ray and neutron data for non-trivial structures (Cassidy *et al.*, 2021 ▸; Mogi *et al.*, 2019 ▸; Spencer *et al.*, 2018 ▸), the user base of the program also consists of scientists inexperienced in the technique who require a simple introduction to the analysis. Other important aspects of high-quality research are reproducibility, repeatability and traceability of results, and proper analysis of parameter un­certainties.

To balance aspects of flexibility and advanced features with ease of access, *GenX*’s key concept is a GUI-based model builder that generates a Python model script. The script can be modified interactively and is executed inside the GUI to produce a model graph and refine a figure of merit (FOM) for a set of parameters. The flexibility of this approach is apparent in the fact that *GenX* can be used to model and fit specular, off-specular or grazing-incidence diffraction data, as well as user-defined functions, with the same interface. This also turned out to be a weakness in the first version of the software since the entrance threshold for new users was too high. In Version 2, a plugin facility was introduced to allow GUI improvements for specific modeling tasks as well as different data loaders. The reflectivity model builder plugin is the most commonly used and can perform the complete script generation for X-ray and neutron reflectivity data in most use cases. This plugin, according to feedback received, lowered the barrier to new users. A materials database plugin further simplifies the parameter definition for known materials and can be used to query online databases for new materials.

In the present third version several new features have been added, in addition to porting the software to Python 3 and wxPython 4. New user models can describe more complex sample structures like element-specific diffusion, magnetic structures propagating through layer boundaries, layer in­homo­geneities or neutron supermirror coatings without the need for manual scripting. A *SimpleReflectivity* interface plugin has been introduced that mimics the simplicity of early analysis tools like *Parratt32* 1 where the sample model is completely described in a single table. With this we hope to lower the entrance barrier for new users of *GenX* still further as the complexity of scripting and the high number of parameters are hidden. More data types have been added for import, and the export and import of results as Open Reflectivity Text (.ort) files (Arnold *et al.*, 2021 ▸) follows the spirit of the FAIR principle (Wilkinson *et al.*, 2016 ▸) that is endorsed by many scientific funding agencies.

The calculation kernel and the capabilities of the models have also been improved. Just-in-time compilation (JIT) for the most calculation-intensive functions is now used to speed up all reflectivity models and allow multi-threaded execution, not just during refinement. GPU computing has also been introduced but only leads to benefits in more complex models, especially when the matrix neutron algorithm is required. For a more rigorous statistical estimate of parameter uncertainties, an interface to the *Bumps* library (Kienzle *et al.*, 2021 ▸) has been developed, together with a dialog reporting such results. As statistical estimates require a suitable χ^2^ FOM we have introduced functionality to include instrument or other experimental errors, and this is described in detail below.

In the following we will report the improvements in more detail, with some general explanations of program use. The reader interested in the software layout of the *GenX* package is referred to the supporting information (Supplement 3) and to the source code. At the time of writing the latest published version of *GenX* is 3.6.12 with roughly 100 000 lines of code.

## 2. User interface

**Figure 1:**
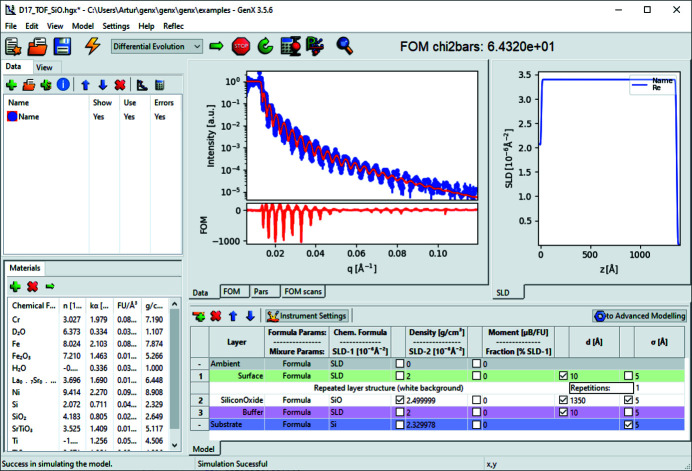
The *GenX* GUI with *SimpleReflectivity* and *SimpleLayer* plugins and widescreen optimized view.

After starting up and loading a model for reflectivity, the *GenX* interface will look similar to the screenshot shown in Fig. 1 ▸. Below the menu and tool bars there is an adjustable area for data sets, data plots and scattering length density (SLD) graphs, a list of known materials, and a table with the *SimpleReflectivity* model. Plugins change the available tabs below the menu bar.

**Figure 2:**
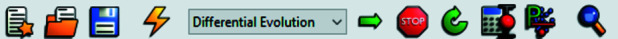
The main program toolbar with elements (from left to right) new model, open model, save model, simulate, select optimizer, start fit, stop fit, restart fit, estimate parameter uncertainty, perform parameter statistical analysis (*Bumps*) and enlarge figures.

The main toolbar (Fig. 2 ▸) hosts the most important actions to load/save a model, simulate and fit, as well as for selecting the used optimizer algorithm and calculating parameter un­certainties. These and additional actions are accessible through the individual menus and most of them have keyboard shortcuts.

### 2.1. Data sets

**Figure 3:**
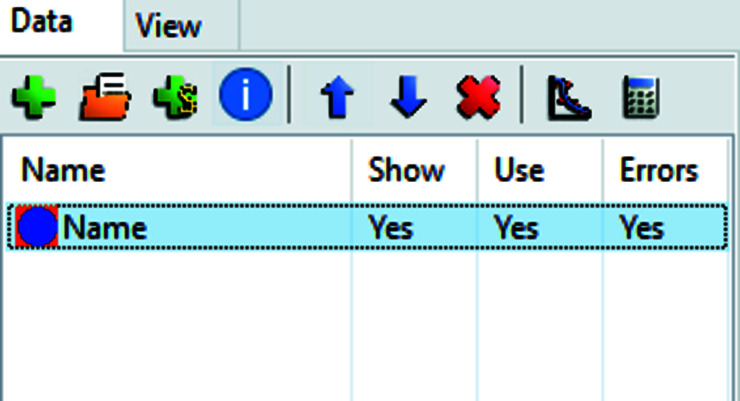
The data set control table, with the toolbar at the top containing elements from left to right: add data set, import data, add simulation, look at metadata information, move up, move down, remove data set, plot options and carry out column calculations. Below the toolbar is the list of data sets with activity status.

Organization for data sets to be fitted is found in the table on the left-hand side of the interface (Fig. 3 ▸). It has a small toolbar for data-specific options and a list of data set entries that can be labeled and selected for plotting and fitting. Data can be imported with *data_loader* plugins that can be selected in the ‘Settings’ menu. Depending on the file format and the data loader used, some or all of the header information is imported and stored within the model following the guidelines for file formats of the Open Reflectometry Standards Organization (ORSO) (Arnold *et al.*, 2021 ▸). The metadata can later be accessed using the ‘Info’ button.

**Figure 4:**
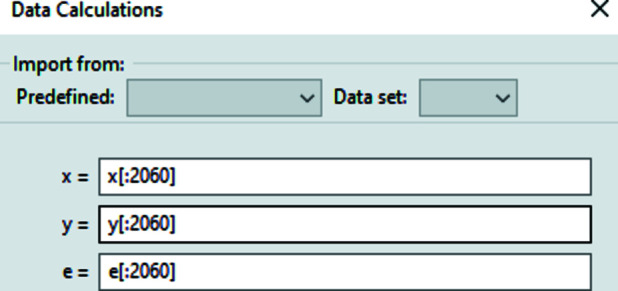
Data column calculations that allow the user to modify the columns of each data set, represented by *NumPy* arrays. In the given example only the first 2060 data points are used.

While, by default, the measured data are used directly in their raw form, the calculation dialog (Fig. 4 ▸) allows modification of the columns using Python expressions. This functionality is most commonly used to filter data points, change the *x*-axis units or implement experimental error calculations, as described below.

When a model is exported to ORSO file format (.ort) all individual data sets with their header information, as well as the model intensity, script and parameters, are written to the file header. This provides an ASCII data format that, at the same time, includes all necessary information to trace the source of the measured data and the sample model used. Data and model can be recovered using the ‘Import’ → ‘New from file’ feature. We therefore encourage authors to include such exported data in any future publication that uses *GenX* for modeling. An example file has been uploaded to Zenodo (https://zenodo.org/record/5770518#.YtpsL3ZBxjE).

### 2.2. Model and fitting

The script that generates the simulation model can be entered in the ‘Script’ tab at the bottom. A script can use any valid Python code but must at least define a simulation function Sim(data) that takes a list of data sets as arguments and returns a list of simulated intensity arrays. For reflectivity models the script is generated automatically by the *Simple­Reflectivity* or *Reflectivity* plugin and can be modified for specific model requirements.

**Figure 5:**
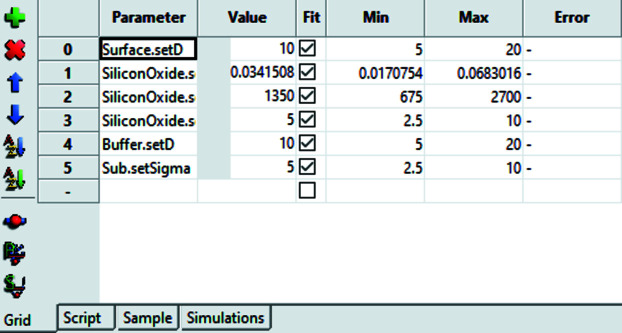
The fitting parameter control grid used in model refinement. The first column is the name of the method used for changing a parameter (setter); in classes of the standard models these are capitalized parameter names with the prefix set. Other columns define the current parameter value, whether it is fixed or fitted, the fit range and parameter uncertainties calculated from the fit. The toolbar on the left has actions (from top to bottom) add parameter, delete parameter, move parameter up, move parameter down, sort class name first, sort method name first, toggle the value slider, project the FOM on the parameter axis and scan the FOM.

Refinement is performed by calling the Sim function and modifying any user-defined parameters from the model. The parameters are defined in the ‘Grid’ tab (Fig. 5 ▸) and are represented by set functions that are called on parameter update (typically parameter setters of the classes representing certain aspects of the model as *e.g.* layers). Starting parameters and minimum and maximum values can be specified in this table, too. Parameter constraints can be implemented by performing calculations within the Sim functions using existing model or user-defined parameter attributes. This allows complex constraints or even mimicking of parameter inequalities (an inequality can be realized by fitting a user parameter in a given fixed range from 0 to 1 and scaling it with the parameter that acts as upper/lower bound).

Changes to the model script and parameters are now realized using the *Command* design pattern, which allows the user to undo previous changes and even remove specific actions from the editing history. An external text editor can also be used to modify the script for more convenient editing of complex models. The temporary file is constantly monitored and the *GenX* model is automatically updated on any change.

### 2.3. Plugin facility

Besides the *data_loader* plugins, *GenX* supports dynamic loading of plugins that modify the GUI functionality. These can be accessed through the ‘Settings’ → ‘Plugins’ menu. Loaded plugins are saved in the *GenX* model file and reloaded when a model is opened. The configuration file, which can be filled from a set of default options, defines which plugins are loaded on program start.

### 2.4. SimpleReflectivity

This plugin allows easy access to reflectometry modeling. A new model is created using a wizard interface that guides the user through generic configuration choices. The sample is then defined within a single table interface that also allows the fit parameters to be chosen. Modeling of X-ray, neutron and polarized neutron measurements is possible, and the sample can have any number of bottom and top layers, as well as a set of central layers that allows repetition in a superlattice. The three stacks that are used to separate bottom, repeated and top layers are color coded as a quick visual hint.

Each layer is defined by a chemical formula with density, by an SLD value (neutron) or as a mixture of two SLDs. Each layer has a magnetization, thickness and roughness. By default, the ‘Grid’ and ‘Script’ tabs are hidden to make the interface more accessible to users without a programming background or *GenX* experience. When the user edits the chemical formula the *SimpleLayer* plugin materials list and the ORSO SLD database (Glavic & ORSO, 2021 ▸) are searched to propose a material density to the user.

At any time the model can be transferred to the *Reflectivity* plugin. This allows modeling to start with a fast and easy interface, and model complexity can be increased later, if necessary.

### 2.5. Reflectivity

This is the traditional plugin for building reflectometry models and also the first plugin that appeared in *GenX 2*. Each component of a sample (substrate and ambient material, stacks and layers) is listed in a specific ‘Model’ tab. Component parameters can be changed with a double click that opens a dialog to enter the values. A similar dialog is available for global sample and instrument parameters. Constraints and user parameters can be added on the ‘Simulation’ tab, which also allows one of the user-defined instruments to be chosen for each data set.

Fitting parameters have to be added by the user, either directly in the ‘Grid’ tab or with a button that is present in the parameter dialog next to each entry. While the interface is more powerful than the *SimpleReflectivity* plugin, it can be overwhelming due to the large number of parameters and the need to edit each layer individually in its separate dialog.

The model generated by the plugin uses specific comment lines to highlight automatically generated lines. The user can change any lines outside these areas for script modifications like complex constraint calculations or to create modifications to existing models.

### 2.6. Other plugins

The current version of *GenX* ships with the following plugins:

*SimpleLayer.* Stores a list of user-defined materials for later use in reflectivity calculations. Materials can be entered with formula and mass density or crystal parameters. The ORSO SLD database (Glavic & ORSO, 2021 ▸) and Crystallography Open Database (Gražulis *et al.*, 2011 ▸) can be used to find data for new materials.

*SpinAsymmetry.* Adds an additional plot that shows the neutron spin asymmetry of data and simulation. Requires that the data set is sorted with alternating spin-up/spin-down channels.

*MagSLD.* Modifies the SLD plot from the *Reflectivity* plugin to show a second *y* axis with magnetic units and the integrated value of the magnetic profile for comparison with macroscopic methods.

*ParameterVault.* Stores model parameter values for later use and comparison. Useful when fitting complex models of many parameters to assist evaluation of possible improvements and revert to old settings.

*Shell.* A Python shell that has access to the program namespace, mostly used for debugging purposes.

*Exporter.* Creates models of other programs from an existing *GenX* reflectivity simulation. Currently only the *BornAgain* software (Pospelov *et al.*, 2020 ▸) is supported.

*SXRD.* A model builder for surface X-ray diffraction that is similar to the *Reflectivity* plugin and shows a 3D image of atomic positions. It can also export the atom positions to .XYZ data files.

## 3. Models

Models included in *GenX* can be directly imported in the model script (as is done by the model-building plugins). The model incorporates both the physical model and a general parameterization of it. Each model and its parameters are described in the dialog accessed through the ‘Help’ → ‘Models help’ menu. All source code can be found in the *GenX* program folder/package directory under genx/models/[model].py. Here we will, for completeness, list all current models and give more detailed descriptions for those not already published.

interdiff. A model for specular and off-specular X-ray reflectivity. See initial publication (Vrugt *et al.*, 2008 ▸).

spec_nx. A combined specular X-ray and neutron model. For specular X-rays it is equivalent to interdiff. Uses *Parratt* (Parratt, 1954 ▸) for X-ray and non-spin-flip neutrons, and *Matrix* (Blundell & Bland, 1992 ▸) for spin-flip calculations. See initial publication (Vrugt *et al.*, 2008 ▸).

spec_adaptive. A model based on spec_nx that allows the user to build complex models of mixing components such as for element-specific interdiffusion. The final SLD profile is generated using adaptive layer segmentation.

spec_inhom. Another spec_nx-based model that simulates superlattices with different inhomogeneities, including macroscopic thickness variation and thickness or roughness variation from bottom to top.

soft_nx. A combined specular X-ray and neutron model equivalent to spec_nx but using SLDs instead of molecular scattering length and density as is common in the soft-matter community.

mag_refl. A model for magnetic resonant soft X-ray reflectivity and specular neutron reflectivity from the same structure, with the possibility of using adaptive layer segmentation or derived roughness factors for the interfaces (Björck *et al.*, 2014 ▸). The model includes both magnetic and structural roughness. The specific X-ray Matrix formalism is described by Stepanov & Sinha (2000 ▸).

sxrd(2). This models surface X-ray diffraction using kinematic diffraction theory. The sxrd2 variant is used by the *SXRD* plugin to build the model. The model itself was added early on (Björck *et al.*, 2008 ▸) and more details of the theory of *SXRD* are provided by Schlepütz (2009 ▸). The model also has an extension for modeling superlattices with the *SUPREX* model (Fullerton *et al.*, 1992 ▸).

### 3.1. Adaptive layer segmentation

The new spec_adaptive model is similar to the standard reflectivity models in terms of model parameters and intensity simulation. In addition to all functionalities from the spec_nx module, a sample can be built of separate *Elements* that can be described independently of each other. This option can be useful to model physical characteristics that are not directly coupled to each other, like a magnetic structure that propagates through a superlattice with incommensurate period or element-specific interdiffusion.

**Figure 6:**
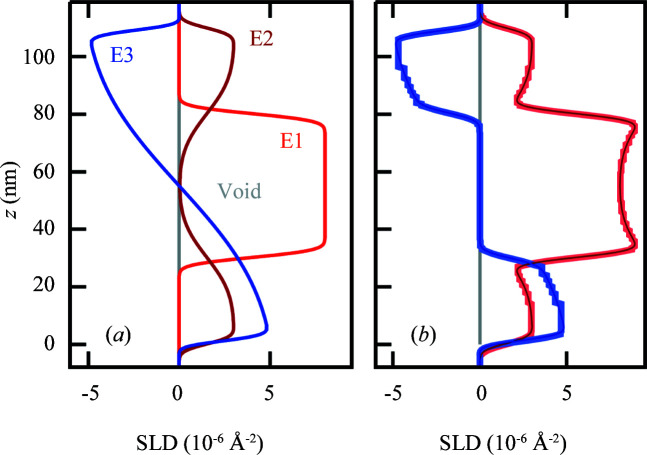
An example of an adaptive layer segmentation model with three *Elements*, E1–E3. The three *Elements* with nuclear SLDs in E1 and E2 and magnetic SLD in E3 are (*a*) shown separately and (*b*) shown in combination and segmentation. The central layer in E1 is defined as a magnetic void, negating the magnetic SLD in this region of the combined model.

Each of the *Elements* is built up from the substrate individually (Fig. 6 ▸). Upon simulation the SLDs for each item are calculated in fine slices (*e.g.* 0.5 Å) and combined. Before the actual simulation, similar total SLD values are merged into one layer (adaptive segmentation) to increase model efficiency. The maximum deviation allowed to combine two layers can be defined by the user for X-ray, neutron and magnetic components. This approach allows the model to define a magnetic roughness that is different from the nuclear structure, magnetic void layers (no magnetization even if other *Elements* define magnetism at this location) and non-Gaussian interface profiles. This model was used by Cassidy *et al.* (2021 ▸) to describe molecular diffusion with mass conservation.

### 3.2. Inhomogeneous superlattices

Superlattices with many repetitions lead to very strong and sharp Bragg peaks that are sensitive to small deviations in the sample structure that would not be noticeable in individual layers. Describing the system with individual layers would generate a model with too many parameters, and constraining these parameters manually would require a large number of parameter couplings. Variation of the layer thickness over macroscopic areas on the sample leads to incoherent averages of reflectivity curves. In this situation the spec_inhom model can be used to change the layer parameters automatically from the bottom to the top of the stack, as well as to perform an incoherent sum over sample thickness variations.

Thickness variation can be modeled using global sample parameters with a Gaussian profile, or different functions like an empirical shape based on pulsed laser deposition plume profiles as described by Glavic (2012 ▸, ch. 3.5.7). Roughness and thickness gradients can either be applied to a complete stack of layers or be defined on a per-layer basis if, for example, interdiffusion only affects one interface in the stack. The roughness increase can be either linear or as the square root of the layer number to model root-mean-square (r.m.s.)-like increases. A gradient for material density can also be applied. An example application of the model can be found in the work of Glavic *et al.* (2016 ▸), modeling thin NdMnO_3_/SrMnO_3_ superlattices with 40 repetitions and varying interface roughness.

Additional stack parameters allow the user to transform the superlattice into a neutron supermirror sequence using either the simplified analytical model from Schelten & Mika (1979 ▸) or an iterative technique (Hayter & Mook, 1989 ▸). This allows the simple definition of model systems with very few parameters describing up to several thousand layers to find the reason for reflectivity deviations or to try new material combinations.

### 3.3. Model performance

Python combined with the *NumPy* library (https://numpy.org/) has established itself as an efficient computing platform for data analysis. *NumPy* can compensate most of the shortcomings that Python, being a interpreted language, has when compared with fast compiled languages like C, C++ or Fortran. Well written *NumPy* implementations are typically two to ten times slower than compiled code, which is sufficient for many applications.

To improve the speed in *GenX* further, we have used *Numba* (Anaconda, 2021 ▸) which uses a JIT compiler to increase the speed of the code. This has been done for parts of the footprint and resolution correction, the reflectivity kernel using the Parratt formula (Parratt, 1954 ▸), the neutron matrix method (Blundell & Bland, 1992 ▸), and expensive complex-number sums for surface diffraction and distorted wave Born approximation calculations for off-specular scattering from roughness. These algorithms have been profiled to use more than 90% of the total computation time. An extension of *Numba* can also deal with GPU computing parallelization using the CUDA programming interface, which is implemented as an alternative. As the possible gain depends strongly on hardware and model complexity the user can choose to activate it. For compatibility, all *NumPy* implementations are still included in the *GenX* libraries and used automatically if *Numba* is not available.

**Figure 7:**
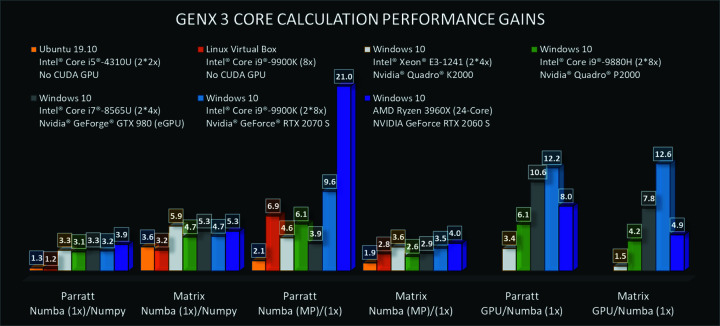
Measured performance gains provided by *Numba* implementation in different configurations. The data used were computed for 4000 data points and a 202 layer sample. Colors represent the computing hardware configuration. The first two blocks represent the improvement from *NumPy* to single-thread *Numba* in the JIT version, the middle two blocks show the improvement when using all available cores compared with single-thread *Numba* and the last two show the gain from single-threaded CPU to GPU implementation.

In Fig. 7 ▸ we show examples of the performance gains for a relatively complex model of 202 layers calculated for 4000 data points on different platforms (all results can be found in the spreadsheet in the supporting information, Supplement 1). This is comparable to real-world fitting problems where *GenX* was tangibly slowed down. The script for running these timings is included with the *GenX* source under tests/numba_performance.py.

The results show that all *Numba* implementations out-perform *NumPy*-based functions even when executed in a single thread. The gain in performance for the neutron matrix method, which is always slower than Parratt’s algorithm, is generally greater. In both cases we also observe that the gain on Linux-based systems is smaller, which we attribute to the generally lower performance of *NumPy* functions on the MS Windows binaries we have used. GPU functions have a significant performance gain compared with the single-core CPU implementation, although the improvement is lower than expected and mostly comparable to using multiple CPU cores.

For model fitting the user has the choice between using a single Python process (multiple *Numba* threads) with CPU or GPU and using the multiprocessing library. The implementation will lower the number of cores used for *Numba* functions for optimal performance and, if GPU computation is chosen, will set aside a single process for GPU calculations while using the CPU cores for all other simulations. Each parameter set of a generation is divided between the processes using asynchronous map functions so that faster processes (like GPU computation) have no unnecessary idle times. If *GenX* is used from the command line, models can be fitted in parallel using the message parsing interface, which is usually available on clusters and supercomputers, providing yet another way of scaling the computations.

## 4. Parameter statistics

The primary reason for fitting models to collected data is to retrieve physical parameters for the measured sample. It is thus important to estimate the uncertainty in the resulting fit parameters. A rough estimation of errors after a *GenX* fit with DE was implemented early on, using the set of parameter values from all generations and filtering those that lead to a limited increase in the FOM (default 5%). This method has the advantage of being applicable to any FOM and giving independent errors for a variety of parameters in positive and negative directions, but it only gives a relative measure and has no rigorous statistical basis. Traditionally, for gradient descent methods like the Levenberg–Marquardt (LM) algorithm (Marquardt, 1963 ▸; Levenberg, 1944 ▸) the uncertainty and covariance estimate are based on the Jacobian matrix calculated at the point of convergence. This technique requires running the algorithm for the final minimization and is implemented in *GenX* when the ‘Calculate errorbars’ action is executed after an LM fit.

**Figure 8:**
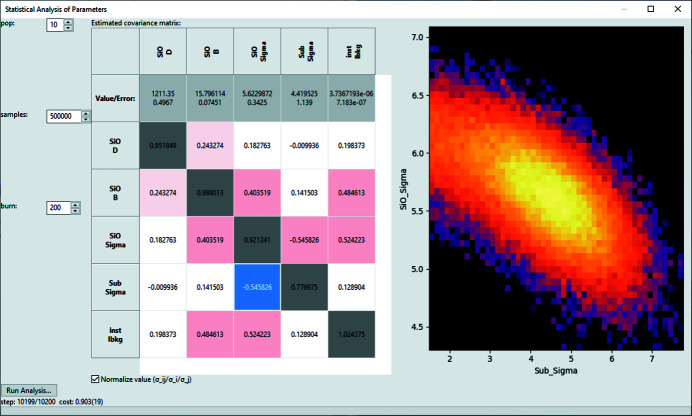
The *GenX* dialog for parameter uncertainty determination. On the left-hand side is the table of parameter values, errors and covariance matrix entries, and on the right is a logarithmic probability plot for a pair of parameters.

A more recent alternative for statistical uncertainty estimation and analysis is based on the likelihood function of parameter ensembles from Markov-chain Monte Carlo (MCMC) simulations (Vrugt *et al.*, 2008 ▸; Schoups & Vrugt, 2010 ▸). An implementation of this technique for model fitting and evaluation is the *Differential Evolution Adaptive Metropolis* (*DREAM*) algorithm that is provided by the *Bumps* library (Kienzle *et al.*, 2021 ▸). It produces estimates of parameter errors and the covariance matrix, and allows probabilities to be visualized for parameter pairs. In *GenX* the estimation can be done after any refinement by generating new MCMC histories or using results from a previous refinement with the *Bumps* optimizer. The ‘Error Statistics’ action will open the dialog shown in Fig. 8 ▸, allowing the user to choose the population size for the MCMC simulation, the number of parameter sets to be used for the estimate and the burn parameter defining the number of iterations to run the algorithm before collecting statistics.

The default values of a population size of 2*N*_pars_ + 2, 10^4^ samples and burn = 200 should be sufficient to estimate uncertainties and possible cross correlations. For publication, an increase in the sample size by a factor of 10 to 100 is encouraged. For the example shown in Fig. 8 ▸ and Table 1 ▸ the default sample size, 50× smaller than in the figure and that could be evaluated in a few seconds, only showed deviations of ∼10%.

Both the Jacobian matrix approach with LM and the MCMC method require the use of error-weighted χ^2^ as the FOM to produce valid uncertainties. For many reflectivity data sets, mostly from X-ray measurements, this can lead to issues with the refinement caused by systematic/instrumental errors from the experiment. *GenX* implements extra functions to include some of these effects in the data analysis, which are described in the following section.

## 5. Experimental errors

It is a well known problem in reflectometry that the information of the measurement is spread over many orders of magnitude in intensity, and this leads to issues in refining to a χ^2^ FOM as relative counting errors can be very different throughout the data set. This issue is very common for X-ray reflectivity, as measurements are often done with the same counting time for each point while the intensity varies over six or more orders of magnitude. In this case, the deviations of the data from a perfect model are dominated by experimental errors rather than the counting statistics. It has therefore been an established practice for these data sets to be fitted using the logarithmic difference without including error bars (Wormington *et al.*, 1999 ▸).

For a rigorous statistical treatment a FOM that does not include data uncertainties is not suitable. We have introduced some functionality into *GenX* to take into consideration the most common experimental errors for existing data sets to solve this problem. Most of these calculations modify the error bars of the data points and are thus, in principle, applicable to any analysis of the data independent of software.

Instrumental errors, often the dominant experimental errors, can be categorized into two classes, global deviations that change the measurement result over many points and point-by-point errors. While the former can best be modeled in the data refinement or corrected during data reduction, the latter lead to actual loss of information, as the data do not contain any correlations that could allow the derivation of the value of the experimental deviation. It is therefore optimal to describe experimental errors with a global impact as special fitting parameters and point-by-point errors as a correction to the χ^2^ calculation. If the model does not yet describe the source of a global error, one can fall back to include these in the error bars to improve the χ^2^ accuracy, but the user needs to be aware of possible issues arising from the correlated nature of these errors.

For reflectometry, assuming that the experiment was carried out correctly, the most common experimental errors are deviations of the reflection angle and deviations from the theoretical footprint correction due to variation over the beam cross section and linear offset of the sample position. We discuss these in detail in the supporting information (Supplement 2) for the example of a laboratory X-ray reflectometer with a nominally square-shaped beam. Most of these considerations are applicable to other instruments or sources of experimental errors as well.

The above-mentioned effects of step error and footprint can be included in *GenX* using three functions that can be used to modify the error values:

rms(*sigmas). Calculates the root-mean-square as 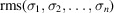 = 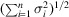 .

dydz(). Returns the numerical derivative from the data points. See the supporting information (Supplement 2) for details.

fpe(xmax, offset=0, inhom=0, steps=10). Returns the footprint error for a given theoretical point of full coverage *x*_max_, the σ of the sample offset relative to beam size and the relative beam inhomogeneity with dominant sub-beams (*N*) as inhom_steps.

In the example SuperAdam_SiO_systematic_errors.hgx that is distributed with *GenX* the total error is calculated using a step error of 6 × 10^−4^°, a sample offset of 10% beam size and 25% inhomogeneity as

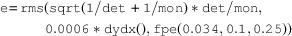




A calibration error in the reflection angle can be fitted using the tthoff instrument parameter with a custom systematic error parameter that can be created similarly to other user parameters:

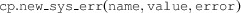




With such corrected standard deviations, a fit conducted with χ^2^ as the FOM yields similar results to a logarithmic refinement. Using the standard counting error does not give satisfactory results – they deviate from the data in the larger half of the *q* range.

## 6. Conclusions

Model flexibility paired with usability have been the key properties that have made *GenX* attractive to the reflectivity and surface scattering communities. We have built on these strengths by adding an even simpler to use interface for reflectivity models, as well as new advanced functionalities for complex model building and statistical analysis. The implementation of error corrections for experimental uncertainties can already improve the quality of the scientific results and we will address experimental issues such as the impact of instrument component imperfections for laboratory and large-scale instruments, as well as sample shape, in more detail in a future publication. We have implemented the first step towards more transparent data analysis according to FAIR principles (Wilkinson *et al.*, 2016 ▸), closely following the recent developments started by the community within ORSO (Arnold *et al.*, 2021 ▸).

With continuous development efforts we hope that *GenX* can continue to be a standard package used for data analysis in this field. The software is developed open source and any contribution from the community is welcome. The code is available at https://sourceforge.net/projects/genx and https://github.com/aglavic/genx.

## 7. Related literature

For further literature related to the supporting information, see D’Agostini (1994 ▸).

## Supplementary Material

Supplement 1: Performance data used to generate Fig. 7. DOI: 10.1107/S1600576722006653/ge5118sup1.xlsx

Supplement 2: Inclusion of experimental errors. DOI: 10.1107/S1600576722006653/ge5118sup2.pdf

Supplement 3: Software layout. DOI: 10.1107/S1600576722006653/ge5118sup3.pdf

## Figures and Tables

**Table 1:** Comparison of error estimation in *GenX* using the three refinement methods, differential evolution (DE), Levenberg–Marquardt (LM) and *Bumps*, on the experimental errors example model SuperAdam_SiO_systematic_errors.hgx

Parameter	*GenX* DE	LM	*Bumps*
SiO *d*	1.2	6.7 × 10^−1^	5.0 × 10^−1^
SiO *b*	2.0 × 10^−1^	5.8 × 10^−2^	7.5 × 10^−2^
SiO σ	7.1 × 10^−1^	2.5 × 10^−1^	3.4 × 10^−1^
Substrate σ	2.1	1.0	1.1
Instrument background	1.9 × 10^−6^	5.6 × 10^−7^	7.2 × 10^−7^

## Footnotes

1 Software for reflectometry from the former Hahn Meitner Institut, now the Helmholtz Center Berlin, no longer maintained.
