# Supplementary material for: GenX 3: the latest generation of an established tool
Source: J Appl Crystallogr. 2022 Jul 30;55(Pt 4):1063–71. doi: 10.1107/S1600576722006653 (PMC9348875; doi:10.1107/S1600576722006653)
Supplement: Supplementary file 2 [file j-55-01063-sup2.pdf]

Supplement 2 - Inclusion of experimental errors

Artur Glavic and Matts Bjork

As the errors discussed in the main article are statistically independent from the counting errors as well as other experimental errors we use the RMS to combine different components. Any experimental errors are treated as normal distributed using the *numpy.random.randn* function to draw random values. Counting errors are simulated from Poisson statistics with *numpy.random.poisson*. The sample model used is a double layer of 15 nm Fe and 10 nm Pt deposited on a Si substrate measured with incident beam of 10^7 counts per point and a constant background level at 10 counts per point.

1 Reflection Angle

For an angle-dispersive measurement the x-position has a global and point-by-point component as there will be a deviation from the perfect calibration of the 0-angle that applies to all data points as well as the step error of the motion stage that will lead to random variations for each data point. While a more complex refinement that includes both x- and y-errors might be possible, we chose to include the offset of a data point into the intensity error by computing the expected intensity change from the slope of the curve:

$$\sigma_y = \sigma_x \frac{\delta y}{\delta x} \tag{1}$$

To estimate values for these errors we use the accuracy and repeatably of a typical rotation stage as 35" and 4", respectively.

Figure S2.1a shows the influence of a random step error on the χ^2 statistics as well as each point component from the average of 100'000 simulations. While a measurement with a perfect instrument (black) yields the expected χ^2 distribution (grey shaded), with slight deviation due to the Poisson statistics of low intensity points, the data with systematic errors (blue) shifts the distribution upwards with individual deviations of up to $100\,\sigma$ in the low angle region where it is expected. Introducing the systematic error into the dataset (red) using equation 1 with numerical derivatives calculated from neighboring data points (eqn. 2) recovers not only the FOM statistics but also the shape of the angular dependent FOM values.

$$\left(\frac{\delta y}{\delta x}\right)_i \overset{numeric}{=} \frac{y_{i+1} - y_{i-1}}{x_{i+1} - x_{i-1}} \tag{2}$$

While this method works best with a dataset of dense points it is generally applicable, as the numeric derivative underestimates the real derivative and thus does not increase the error values of the dataset above what is justified from the systematics. The results in figure S2.1ab also illustrate why the standard χ^2 FOM is not suitable to refine models to such datasets as the weights in the range of 2Θ between 1° and 3° outweighs any deviation of the model to the data at higher angles, loosing the information in that range.

Treating the calibration error with the same formalism does recover the average χ^2 but not its distribution as can be seen in figure S2.1b. This shows that inclusion of such global instrument effects as a model parameter is better suited than to alter the error bars.

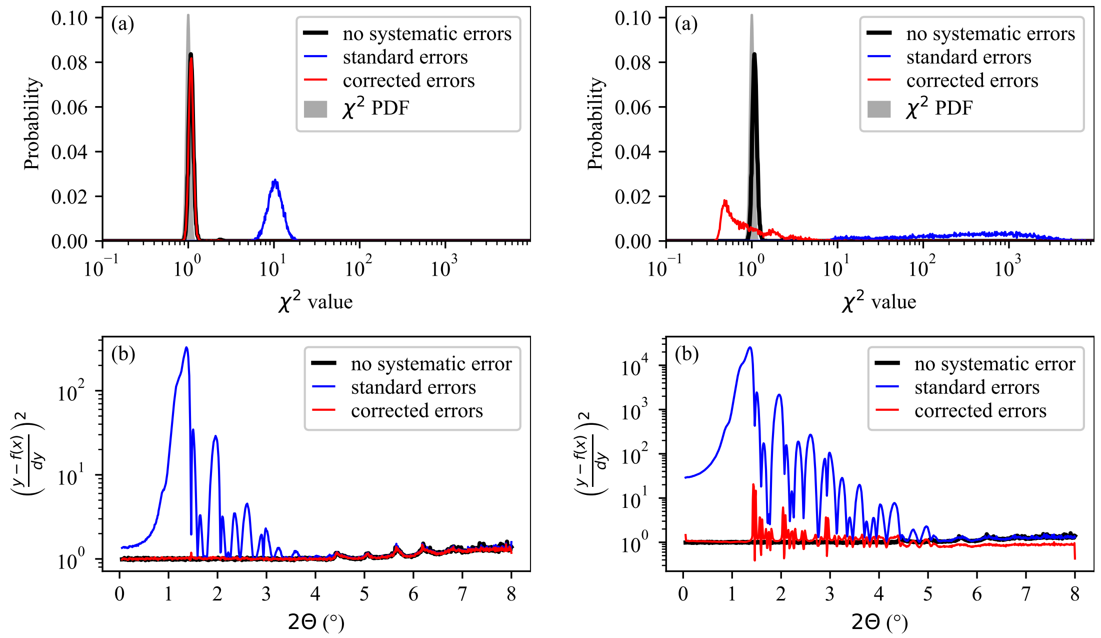

(a) step-error of the 2Θ angle

(b) calibration error of the 2Θ angle

Figure S2.1: χ^2 statistics of simulation of random experimental errors and influence of applying the corrections described in the main text on the error bars of the dataset.

2 Intensity Inhomogeneity

As most samples are smaller than the projected beam size at the starting angle of a reflectivity measurement, angular-dispersive measurements commonly require footprint correction. This correction can either be applied directly to the data or included in the model simulation. In both cases, the correction assumes a certain beam cross section profile that will be idealized with respect to the actual beam that was used. The influence of deviation from the ideal beam shape is non-trivial as it not only depends on the amount of deviation but the specific length scale of that variation. At the same time, the intensity at the critical edge is high and thus statistical errors are negligible.

To estimate the influence of such intensity profile variation we consider a finite set of beam contributions that make up the actual beam cross section and compare it to an ideal square shaped beam. Figure S2.2a shows a sketch of the used model with a number of sub-beams N and the number of such sub-beams covering the sample at a certain angle n. For a centered sample, each sub-beam n is made up of two components i that are on opposite sites of the beam center. Each sub-beam has an average intensity of $1/N$ with a relative variation of the systematic error $\sigma_{rel} = \sqrt{2}\,\sigma_i$. The total intensity of a beam can be measured with high precision, we therefore consider the total intensity to always be normalized to 1.

$$I_i = \frac{1+\Delta_i}{N} \quad \text{with} \quad \mu_\Delta = 0 \quad \& \quad \sigma_\Delta = \sigma_{rel} \tag{3}$$

$$I(n) = \sum_{i=1}^{n} \frac{I_i}{C} \quad \text{with} \quad C = \sum_{i=1}^{N} I_i \approx 1 \tag{4}$$

$$I(n) = \frac{\sum_{i=1}^{n} I_i}{\sum_{i=1}^{N} I_i} = \frac{\sum_{i=1}^{N} I_i - \sum_{i=n+1}^{N} I_i}{\sum_{i=1}^{N} I_i} = 1 - \frac{\sum_{i=n+1}^{N} I_i}{\sum_{i=1}^{N} I_i} \tag{5}$$

With the normalization criterion (4) conventional error propagation does not apply for $I(n)$ as the errors on the normalization parameter C are correlated with the errors in the numerator. We therefore derive the errors for the limiting cases of small n and large n where the two descriptions in (5) have much shorter sums in the numerator that dominate over the error in the denominator. From this we can derive two relationships for errors on the measured intensity:

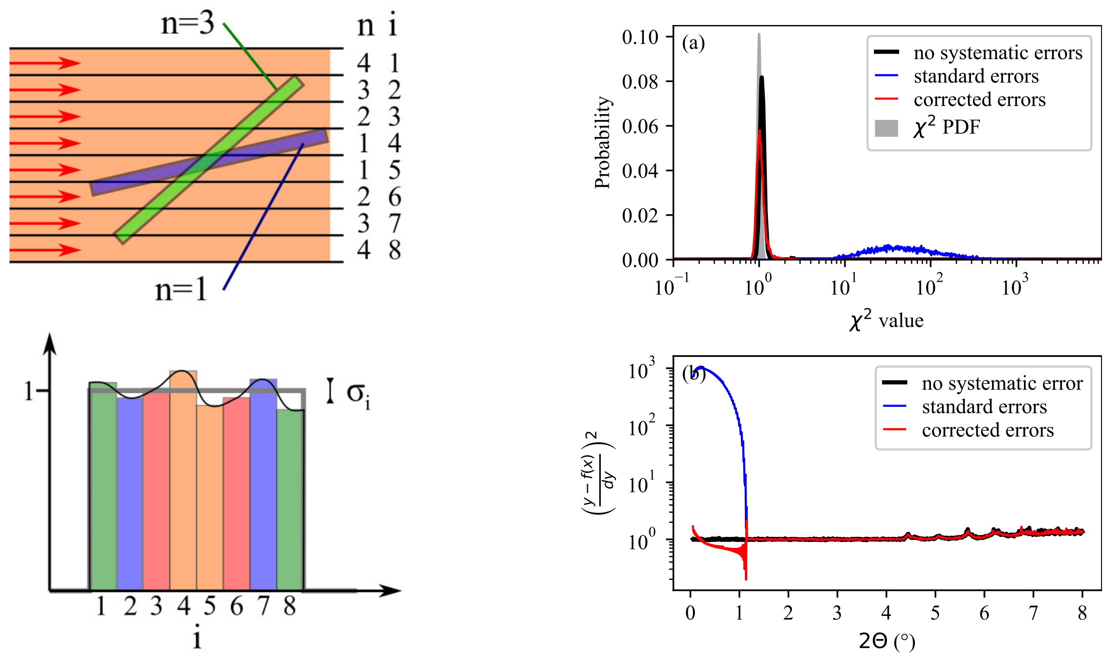

(a) Sketch of the intensity deviation that originates from beam cross section variations. The individual contributions with random variation i and the beam covering the sample for a given angle n are denoted.

(b) Statistics of simulation of random beam cross section inhomogeniety on χ^2 and influence of applying the corrections described in the main text on the error bars of the dataset.

Figure S2.2: Analysis of beam inhomogeniety.

$$\sigma_{I(n)} \overset{n\approx 1}{\approx} \sqrt{\sum_{i=1}^{n}\left(\frac{\sigma_{rel}}{N}\right)^2} = \frac{\sqrt{\sum_{i=1}^{n}\sigma_{rel}^2}}{N} = \frac{\sigma_{rel}\sqrt{n}}{N}$$

$$\frac{\sigma_{I(n)}}{I(n)} \approx \frac{\sigma_{rel}\sqrt{n}}{N} \Big/ \frac{n}{N} = \frac{\sigma_{rel}}{\sqrt{n}} \tag{6}$$

$$\sigma_{I(n)} \overset{n\approx N}{\approx} \sqrt{\sum_{i=n+1}^{N}\left(\frac{\sigma_{rel}}{N}\right)^2} = \frac{\sqrt{\sum_{i=n+1}^{N}\sigma_{rel}^2}}{N} = \frac{\sigma_{rel}\sqrt{N-n}}{N}$$

$$\frac{\sigma_{I(n)}}{I(n)} \approx \frac{\sigma_{rel}\sqrt{N-n}}{N} \Big/ \frac{n}{N} = \frac{\sigma_{rel}\sqrt{N-n}}{n} = \sigma_{rel}\sqrt{\frac{N}{n^2} - \frac{1}{n}} \tag{7}$$

Using the mean intensity $I(n) \approx \frac{n}{N}$ for the approximation in equations (6) and (7). We approximate the total error by linear interpolation between the limiting cases:

$$\frac{\sigma_{I(n)}}{I(n)} \approx \sigma_{rel}\left(\frac{N-n}{N-1}\frac{1}{\sqrt{n}} + \frac{n-1}{N-1}\sqrt{\frac{N}{n^2} - \frac{1}{n}}\right) \tag{8}$$

There are two important statements that can be made from equation (8); The impact of the footprint error is largest for small angles (small n) and for large N (high frequency variation) the errors decrease rapidly for finite angles. Therefore the impact on the χ^2 is mostly on the total value and less on the range of q where structural information resides. It should also be clear, that a quantitative incorporation of the error would require detailed beam characterization, which is often not practical.

We have done numerical simulations of a footprint error by using $2N$ random varying intensity points that are spline interpolated and integrated to retrieve the measured intensity. The effect was calculated for 20 steps from $N = 2$ to $N = 22$ and then corrected with equation (8) using $N = 10$. Results of these simulations are shown in figure S2.2b). The corrected errors mostly recover the χ^2 distribution

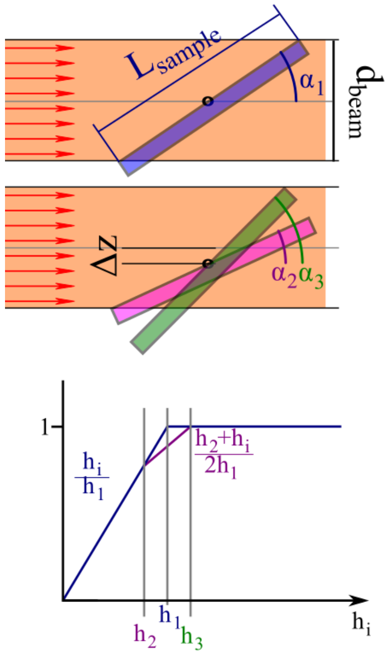

(a) Sketch of the model for measured intensity deviation that originates from a linear sample offset. The slope of the reflected intensity changes at the angle α_2 where the edge of the beam reaches the outer side of the sample until the full sample is covered at α_3.

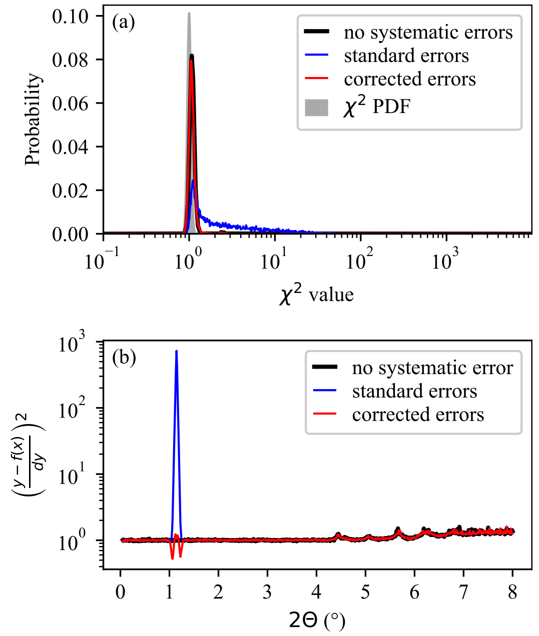

(b) Statistics of simulation of random sample position offset on χ^2 and influence of applying the corrections described in the main text on the error bars of the dataset.

Figure S2.3: Analysis of sample position offset.

but slightly overcorrect points at larger sample coverage. Although the correction only uses one value of N it can practically be used for this estimation. As the critical edge range does not have much influence on relevant model parameters a slight over correction is acceptable. An alternative solution could be to filter the low angle region for FOM calculations to estimate parameter uncertainties.

3 Sample Offset

Another common deviation from the perfect beam profile is caused by a linear offsets of the sample position from the center of the beam. In contrast to the theoretical linear increase until the angle of full coverage there is a decrease in slope when the edge of the beam reaches one side of the sample until the full sample is covered. This leads to the deviation of intensity defined by the linear sample offset Δz:

$$\frac{I(\alpha_i, \Delta z)}{I_0} = \begin{cases} 1 & \text{for} \quad h_3 \leq h_i \\ \frac{h_2+h_i}{2h_1} & \text{for} \quad h_2 \leq h_i < h_3 \\ \frac{h_i}{h_1} & \qquad\qquad h_i < h_2 \end{cases} \tag{9}$$

$$\text{with} \quad h_j = \sin\alpha_j \quad ; \quad h_1 = \frac{d_{beam}}{L_{sample}} \quad ; \quad h_{2/3} = h_1\left(1 \mp \frac{2\Delta z}{d_{beam}}\right) \tag{10}$$

The deviation from the ideal alignment with $\Delta z = 0$ is therefore, introducing the relative deviation $s_i = \frac{h_i}{h_1} - 1$ and offset $\frac{\Delta z}{d_{beam}} = \Delta h$:

$$\Delta I(s_i, \Delta h) = \frac{I(\alpha_i, \Delta z) - I(\alpha_i, 0)}{I_0} = \begin{cases} 0 & \text{for} \quad \Delta h \leq s_i \\ \frac{-\Delta h + |s_i|}{2} & \text{for} \quad -\Delta h \leq s_i < \Delta h \\ 0 & \qquad\qquad s_i < -\Delta h \end{cases} \tag{11}$$

Deviation is only found for the range between $s_i = \pm\Delta h$, which depends on the actual sample offset and is largest at $s_i = 0$ with $\Delta I = -\frac{\Delta h}{2} = -\frac{\Delta z}{2d_{beam}}$. To determine the error of the intensity $\sigma_{I(s_i)}$ for a

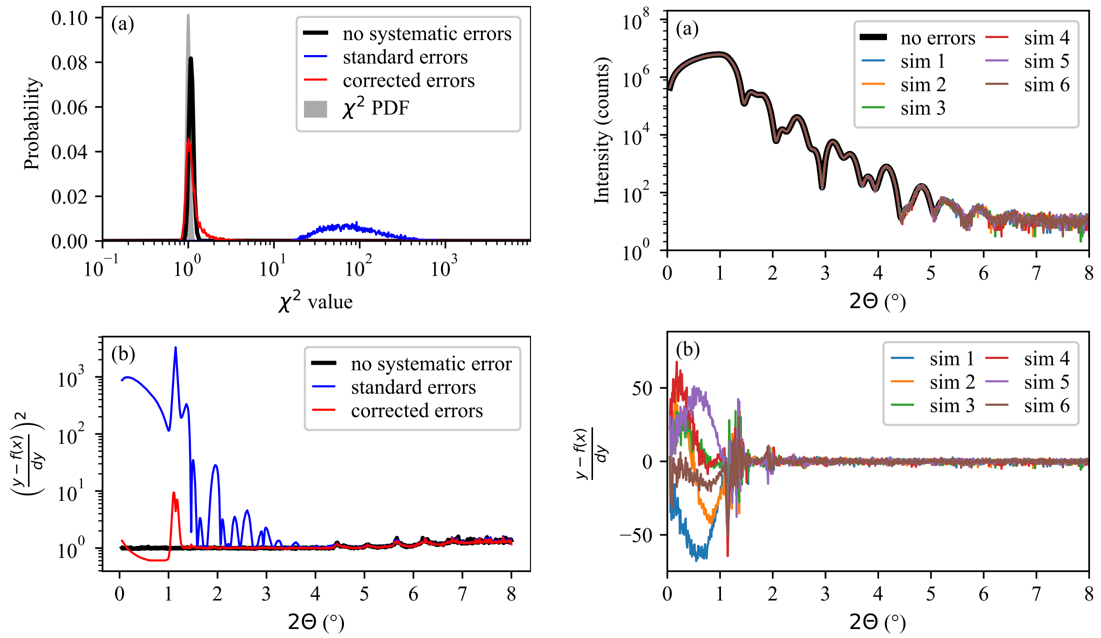

(a) χ^2 statistics and relative deviations

(b) Set of individual simulations and their error weighted deviations

Figure S2.4: Simulation of random step-error, beam inhomogeniety and sample offset as well as influence of applying the corrections described in the main text on the error bars of the dataset.

given location s_i one has to consider the intensity distribution that follows from a given sample offset distribution $\sigma_h = \frac{\sigma_z}{d_{beam}}$. For Gaussian distributed sample offsets this can be derived by calculating the integral for the variance in three regions below, inside and above $|s_i| <= \Delta h$ (with Gaussian distributions $G(x, \sigma)$):

$$\sigma^2_{I(s_i)} = \int_{\Re} \Delta I(s_i, x)^2 G(x, \sigma_h)\, dx \tag{12}$$

$$= \int_{-\inf}^{-|s_i|} \Delta I(s_i, x)^2 G(x, \sigma_h)\, dx + \int_{-|s_i|}^{|s_i|} 0\, dx + \int_{|s_i|}^{\inf} \Delta I(s_i, x)^2 G(x, \sigma_h)\, dx \tag{13}$$

$$= 2 \cdot \int_{|s_i|}^{\inf} \Delta I(s_i, x)^2 G(x, \sigma_h)\, dx \tag{14}$$

$$= \int_{|s_i|}^{\inf} (x - |s_i|)^2 G(x, \sigma_h)\, dx \tag{15}$$

The integral in equation (15) corresponds to the variance of a truncated Gaussian distribution, which can be calculated using a combination of Gauss and error functions. Unfortunately the calculation requires differences of close numbers and the floating point precision of the error function failed for deviations of s_i larger than $\approx 5\sigma_h$. We have therefore introduced an approximation of this function that fit the parameter distribution:

$$\sigma^2_{I(s_i)} \approx \frac{\sigma_h}{2} e^{-\frac{|s_i|}{\sigma_h}} \tag{16}$$

Using this systematic error contribution for the χ^2 calculation the original distribution can be recovered nearly perfectly as shown in figure S2.3b.

4 Combined Systematics

Assuming the angular offset is treated as a fit parameter we have combined the influence of step error, intensity variation and sample offset into one simulation and applied all above corrections. Figure S2.4a

shows the combined influence onto the FOM with a correction applied using the RMS of counting and all systematic errors. The described corrections recover the χ^2 distribution and quadratic deviations very well and thus allows fitting and parameter error analysis using this FOM. Figure S2.4b illustrates the effects on a set of random simulations used for the analysis. Notably, none of the systematic deviations that dominate the regular χ^2 parameter are even visible in the measured data.

To include these effects the dataset calculation *GenX* introduces three functions that can be used to modify the error values:

`rms(*sigmas)` Calculates the root mean squared as $rms(\sigma_1, \sigma_2, ..., \sigma_n) = \sqrt{\sum_{i=1}^{n} \sigma_i^2}$

`dydz()` Return the numeric derivative from the data points according to equation (1)

`fpe(xmax, offset=0, inhom=0, steps=10)` Returns the footprint error for a given theoretical point of full coverage xmax, sample offset relative to beam size and relative beam inhomogeneity with dominant sub-beams (N) as inhom_steps.

In the example "SuperAdam_SiO_systematic_errors.hgx" that is distributed with *GenX* the total error is calculated using a step-error of $6 \cdot 10^{-4\circ}$, sample offset of 10% beam size and 25% inhomogeneity as:

```
e=rms(sqrt(1/det + 1/mon) * det/mon, 0.0006 * dydx(), fpe(0.034, 0.1, 0.25))
```

With such corrected standard deviations, a fit conducted with χ^2 as FOM yields similar results to a logarithmic refinement. Using the standard counting error does not yield satisfactory results that deviate from the data at the larger half of the q range.

5 Fit Parameter for Systematic Errors

As described above, the correlation of errors across the whole data range, as is the case for angular calibration errors, is not described by increased error bars very well. In addition, better fit parameters might be derived by describing such errors within the model and fitting the actual offset together with the model. Unfortunately, this approach can also distort the results if there are cross-correlations between the instrument parameter and one or more model parameters.

To counteract this effect, *GenX* 3 introduces a specific type of user parameter that adds a penalty factor to the FOM that increases with the square of the parameter (p_l) deviation from its expected value (μ_{p_l}):

$$\chi^2_{FOM} = \sum_k \frac{(y - f(x))^2}{\sigma_k^2} + \sum_l \frac{(p_l - \mu_{p_l})^2}{\sigma_{p_l}^2} \tag{17}$$

This approach is analogous to a commonly applied method in other fields like particle physics to deal with normalization errors [1]. Such parameter is generated in *GenX* similar to conventional user parameters with on additional argument for the systematic standard deviation:

```
cp.new_sys_err(name, value, error,
weight=1.0, correction=0.0)
```

The FOM penalty can optionally be scaled with the *weight* parameter and a linear offset, *correction*, can be added.

References

[1] G. D'Agostini. On the use of the covariance matrix to fit correlated data. *Nuclear Instruments and Methods in Physics Research Section A: Accelerators, Spectrometers, Detectors and Associated Equipment*, 346(1-2):306–311, jul 1994.
