# Supplementary material for: GenX 3: the latest generation of an established tool
Source: J Appl Crystallogr. 2022 Jul 30;55(Pt 4):1063–71. doi: 10.1107/S1600576722006653 (PMC9348875; doi:10.1107/S1600576722006653)
Supplement: Supplementary file 3 [file j-55-01063-sup3.pdf]

Supplement 3 - Software Layout

Artur Glavic and Matts Bjork

The GenX program is contained within a single Python package grouped into sub-packages and that can be installed via PyPI using *pip install genx*3 or directly from the source distribution. Sub-packages are used to organize the modules between the generic GenX functionality (*genx*, *genx.core*, *genx.remote*), physical models (*genx.models*), data loader and extension plugins (*genx.plugins*) and the WX-based graphical user interface (*genx.gui*). While the physical models could be used independent of other GenX functionality, we expect that users will access them through the general GenX model architecture by either using the *genx.api* scripting interface or the GUI.

Figure S3.1 illustrates how this architecture is structured. The central *Model* object contains a model script as well as the fitting parameters and dataset containing datastructures. The script can be any valid Python code but generally will import one of the supplied models and define the model parameters using associated data classes (currently GenX specific but we are in the process of porting them to Python dataclasses). Any model script has to define a function called *Sim* that is called with the list of datasets to calculate the model intensity. When a simulation is triggered the model script is executed to dynamically create a Python module with a local namespace that includes the *Sim* function and all parameter class instances. In the next step the setter methods defined in the parameter table are executed with the current value for each fit parameter. Finally the *Sim* function is called with the list of datasets to generate y-values for the x-array of each dataset.

For refinement of model parameters a *GenxOptimizer* derived class re-uses the virtual module, modifies the parameter values and re-executes the setter and *Sim* calls to generate the intensity values for each set of parameter values. A figure of merit (FOM) function is called with the simulated and measured values as well as errorbars to calculate the FOM for a parameter set. For the differential evolution (*DiffEv*) and *BumpsOptimizer* classes the model can also be executed on multiple processes (*multiprocessing* library). Each process compiles its own *GenXModelScript* and only the parameter sets and resulting intensity values are transferred to/from the main thread.

Both the *Model* and *GenxOptimizer* objects are controlled by a central *ModelController* class that handles various control functionalities like thread communication, saving and loading as well as keeping track of a change history that can be used from the GUI for undo/redo actions. Both the GUI and *gnex.api* user interfaces work together with the *ModelController* and data loader plugins to form the functional GenX environment. The full functionality provided by the GUI interface does not directly modify the functional elements but rather sits on-top to provide convenient interactivity.

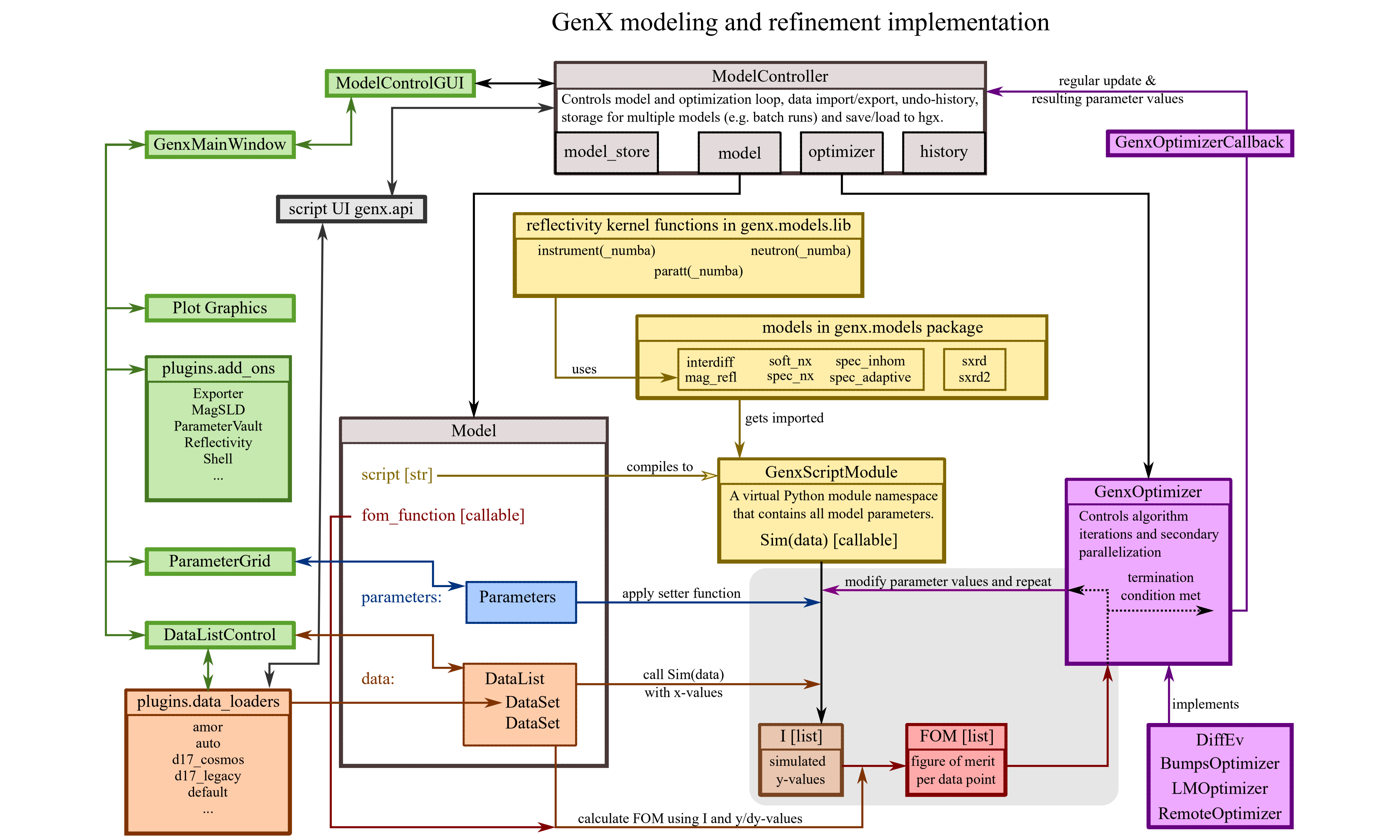

Figure S3.1: Sketch of the high-level architecture of GenX. The green boxes represent different GUI functionalities. The yellow boxes represent hierarchy of the models. Purple represent optimizer building blocks.
